# Congenital adrenal hyperplasia- presenting as central precocious puberty

**DOI:** 10.1186/1687-9856-2013-S1-P118

**Published:** 2013-10-03

**Authors:** Vimal Mavila Veetil, MC Naseerali

**Affiliations:** 1 MIMS, India

## Aims

To demonstrate the presentation of Congenital adrenal hyperplasia (CAH) as central precocious puberty.

## Materials and methods

4 children with mean age of 6.5 (5.5, 6.5, 6.5, 7.5) who presented to the out patient department with precocious puberty between December 2008 to December 2011were studied.

## Results

3 were boys, out of which 2 were twins. 3 boys were diagnosed to have CAH after presentation to the OPD. They had mean bone age of 12 years. They had elevated testosterone (mean : 2.4 ng/ml) and 17 hydoxyprogesterone ( mean : 24 ng/ml ) at presentation. They had clinical (testicular volume 5ml) and biochemical (mean basal LH : 6 ng/ml )evidence of central precocious puberty. The girl was already diagnosed to have CAH at birth itself, but was on irregular treatment. She presented with menarche at the age of 7.5 and had basal LH of 8 ng/ml. All the patients were started on replacement with hydrocortisone, fludrocortisones and GnRH analogue ( Leuprolide depot ).

## Conclusions

Central precocity may be due to undiagnosed CAH . Improperly treated CAH may also lead to central precocious puberty. Treatment may involve GnRH analogues along with adrenal steroids.

